# National Outbreak of *Acanthamoeba* Keratitis Associated with Use of a Contact Lens Solution, United States

**DOI:** 10.3201/eid1508.090225

**Published:** 2009-08

**Authors:** Jennifer R. Verani, Suchita A. Lorick, Jonathan S. Yoder, Michael J. Beach, Christopher R. Braden, Jacquelin M. Roberts, Craig S. Conover, Sue Chen, Kateesha A. McConnell, Douglas C. Chang, Benjamin J. Park, Dan B. Jones, Govinda S. Visvesvara, Sharon L. Roy, for the Acanthamoeba Keratitis Investigation Team

**Affiliations:** Centers for Disease Control and Prevention, Atlanta, Georgia, USA (J.R. Verani, S.A. Lorick, J.S. Yoder, M.J. Beach, C.R. Braden, J.M. Roberts, D.C. Chang, B.J. Park, G.S. Visvesvara, S.L. Roy); Illinois Department of Public Health, Chicago, Illinois, USA (C.S. Conover); California Department of Public Health, Sacramento, California, USA (S. Chen); Florida Department of Health, Tallahassee, Florida, USA (K.A. McConnell); and Baylor College of Medicine, Houston, Texas, USA (D.B. Jones)

**Keywords:** Acanthamoeba, keratitis, contact lens solution, contact lenses, cornea, disease outbreak, parasites, protozoa, United States, research

## Abstract

An outbreak of *Acanthamoeba* keratitis, a rare, potentially blinding, corneal infection, was detected in the United States in 2007; cases had been increasing since 2004. A case–control study was conducted to investigate the outbreak. We interviewed 105 case-patients from 30 states and 184 controls matched geographically and by contact lens use. Available contact lenses, cases, solutions, and corneal specimens from case-patients were cultured and tested by molecular methods. In multivariate analyses, case-patients had significantly greater odds of having used Advanced Medical Optics Complete Moisture Plus (AMOCMP) solution (odds ratio 16.9, 95% confidence interval 4.8–59.5). AMOCMP manufacturing lot information was available for 22 case-patients, but none of the lots were identical. Three unopened bottles of AMOCMP tested negative for *Acanthamoeba* spp. Our findings suggest that the solution was not intrinsically contaminated and that its anti-*Acanthamoeba* efficacy was insufficient. Premarket standardized testing of contact lens solutions for activity against *Acanthamoeba* spp. is warranted.

## Abstract 2

Premarket standardized testing for *Acanthamoeba* spp. is warranted.

*Acanthamoeba* keratitis (AK), a painful corneal infection that may lead to vision loss or enucleation, is caused by the ubiquitous free-living *Acanthamoeba* spp. (*1**–**4*). AK occurs primarily among users of soft contact lenses (*5*), with an estimated US annual incidence of 1–2 cases per million contact lens users (*6*).

In May 2006, the Centers for Disease Control and Prevention (CDC) was contacted by the Illinois Department of Public Health regarding a possible increase in AK cases in the Chicago area during the preceding 2 years. Investigators at the University of Illinois at Chicago were conducting a case–control study to identify possible risk factors. In October 2006, CDC informally surveyed ophthalmologists across the country to ascertain whether cases of AK were increasing elsewhere; results were inconclusive. In January 2007, CDC initiated a retrospective survey of 22 ophthalmology centers nationwide. By early March 2007, results obtained from 10 centers in 9 states showed a rise in the number of culture-confirmed cases during 2004–2006 compared with 1999–2003.

On March 16, 2007, we initiated a national outbreak investigation. On May 23, a preliminary analysis compared data from the first 46 interviews of patients with culture-confirmed AK, with data obtained from 126 healthy adult contact lens users ascertained in a 2006 national outbreak investigation of *Fusarium* keratitis (*7*). The analysis indicated that the odds of having ever used Advanced Medical Optics Complete MoisturePlus (AMOCMP) multipurpose contact lens solution were 20× greater for AK case-patients than for controls. These results were communicated to the Food and Drug Administration (FDA) and were rapidly disseminated (*8*). On May 26, 2007, the company voluntarily recalled AMOCMP from domestic and international markets. Although public health action was taken on the basis of the preliminary analysis, we report here the results of a matched case–control study designed to verify the findings of the preliminary analysis, to identify additional risk factors for AK, and to guide recommendations to prevent future cases.

## Methods

### Case Definition and Case Finding

Case-patients had been given a diagnosis of AK by an ophthalmologist; had symptom onset on or after January 1, 2005; and had *Acanthamoeba* spp. identified from cultures of corneal specimens. Requests to report AK cases were disseminated through CDC’s Epidemic Information Exchange system and through ophthalmology and optometry electronic mailing lists; websites; and associations at the national, state, and local levels. We also queried several referral microbiology laboratories and ophthalmology centers to find cases. Cases included in a concurrent study by University of Illinois at Chicago investigators were excluded (*9*).

### Case-Patient Data Collection and Laboratory Investigation

We used standardized questionnaires to interview case-patients by telephone to obtain demographic characteristics, information regarding illness, contact lens–related product use, and hygiene practices and behavior during the month before symptom onset. An Internet-based visual aid was available to assist with specific product recognition. Ophthalmologists who were treating case-patients provided information by telephone- or self-administered questionnaires regarding diagnostic methods, treatment, and clinical outcomes.

Available clinical specimens (e.g., corneal scrapings or biopsy specimens, *Acanthamoeba* culture isolates) and environmental samples (e.g., opened and unopened contact lens solution bottles, lenses, lens cases) were sent to CDC laboratories. Specimens were processed for *Acanthamoeba* spp. by culture (*4*) and molecular analysis (*10*), including genotyping (*11*).

### Case–Control Study

All interviewed case-patients were eligible. Control subjects had no history of AK and were >12 years of age. We attempted to match 3 controls to each case-patient by contact lens use (i.e., soft lenses, rigid lenses, or no contact lens use) and by geographic location using a reverse address directory to identify controls who resided near each case-patient. Because rigid lens use is uncommon, we did not attempt to obtain geographically matched controls for this group. Controls completed a standardized, telephone-administered questionnaire that asked about behavior and product use during the 1 month before their matched case-patient had symptom onset.

### Data Analysis

Data were double-entered by using Visual FoxPro 8.0 (Microsoft Corp., Redmond, WA, USA) and analyzed by using SAS 9.1 (SAS Institute Inc., Cary, NC, USA). Conditional logistic regression was used to estimate odds ratios (ORs) and 95% confidence intervals (CIs) for univariate and multivariate analyses; significance was defined as p<0.05. All variables that were significantly associated with AK by univariate analysis were further investigated by using a multivariate model.

## Results

### Descriptive Epidemiology

**Figure 1:**
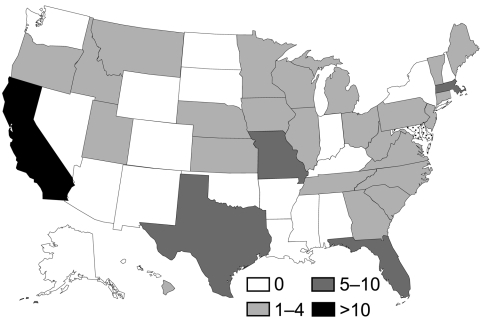
*Acanthamoeba* keratitis case-patients by state, USA (N = 105). *Number of interviewed case-patients per state. Because of incomplete case reporting and enrollment in case–control study, incidence rates were not calculated.

Case-patients were enrolled during March 16–July 10, 2007. Of 221 AK patients identified from 37 states and Puerto Rico, 158 (71%) had infections that met the case definition. We interviewed 105 (66%) case-patients from 30 states (Figure 1) and the treating ophthalmologists of 92 case-patients (88%).

**Figure 2:**
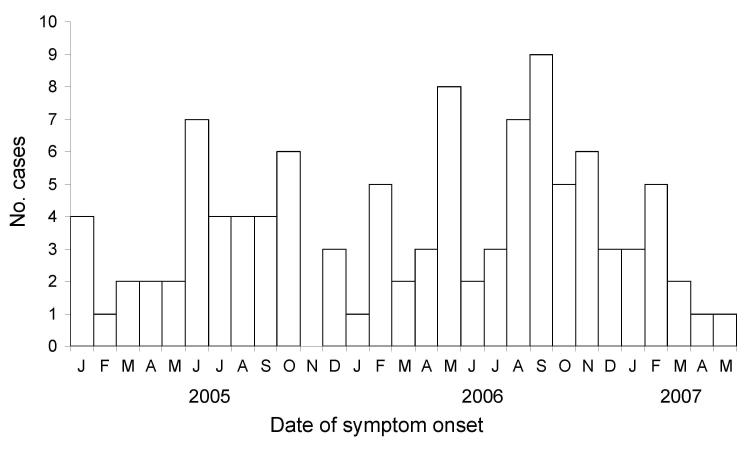
Symptom onset of cases of *Acanthamoeba* keratitis, by month and year, United States, 2005–2007 (N = 105).

Times of symptom onset (Figure 2) did not show any obvious trends or a single period of peak exposure. The 105 case-patients were predominantly female (67 [64%]) with a median age of 29 years (range 12–76 years) (Table 1). Of these case-patients, 93 (89%) wore contact lenses (82 [88%] used soft contact lenses), and 87 (94%) reported using some type of cleaning or disinfecting solution (78 [90%]) used a multipurpose solution). Of the 6 contact lens users who did not report use of any cleaning or disinfecting solution, 1 used daily disposable lenses, 1 used extended-wear lenses that were replaced with no cleaning, 2 used only saline solution, 1 used saline solution and rewetting drops, and 1 did not recall which types or brands of solutions were used.

**Table 1:** Demographic and clinical characteristics of 105 patients with *Acanthamoeba* keratitis, United States, 2005–2007

Characteristic	No. (%)
Sex	
Female	67 (64)
Age, y*	
12–17	27 (26)
18–24	17 (16)
25–34	15 (14)
35–49	24 (23)
>50	22 (21)
Contact lens wear	
Did not use contact lenses	12 (11)
Used contact lenses	93 (89)
Lens type	
Soft lenses	82 (88)
Rigid lenses	10 (11)
Hybrid lenses	1 (1)
Contact lens solution use among contact users†	
Did not use cleaning or disinfecting contact lens solution	6 (6)
Used any type of cleaning or disinfecting contact lens solution	87 (94)
Type of solution used§	
Multipurpose solution	78 (90)
Hydrogen peroxide solution	6 (7)
Daily cleaner	11 (13)
Affected eye	
Right	53 (50)
Left	44 (42)
Both	8 (8)
Symptoms when treatment sought‡§	
Pain	78 (74)
Redness	78 (74)
Sensitivity to light	76 (72)
Sensation of foreign body	71 (68)
Increased tearing	59 (56)
Blurred vision	57 (54)
Discharge from eye	20 (19)
Clinical status¶	
Resolved with pharmacologic therapy	32 (38)
Currently receiving pharmacologic therapy	29 (34)
Corneal transplant performed	21 (25)
Corneal transplant planned	3 (4)
Most recent visual acuity with best correction in affected eye#	
20/20	17 (24)
20/25–20/100	24 (34)
20/>200	29 (41)

*Median 29 y, range 12–76 y. †During 1 month before illness onset; n = 93. ‡Not mutually exclusive. §Median time from symptom onset to anti-Acanthamoeba therapy (n = 80) was 49 d (range 4–197 d). ¶At the time of treating ophthalmologist interview; n = 85. #At the time of treating ophthalmologist interview; n = 70.

The most frequently reported symptoms among case-patients were pain, redness, sensitivity to light, and sensation of a foreign body (Table 1). The median time from onset of symptoms to initiation of anti-*Acanthamoeba* treatment was 49 days, range 4–197 days. At the time of their ophthalmologist interview, 24 (28%) of 85 had either undergone or were awaiting a corneal transplant, and 29 (41%) of 70 had a visual acuity of 20/200 or worse with best correction (i.e., legally blind) in the affected eye.

### Case–Control Study

**Figure 3:**
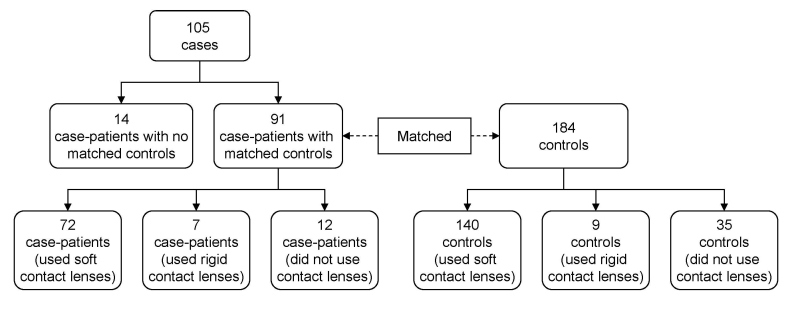
Matching of case-patients with *Acanthamoeba* keratitis and controls, United States, 2005–2007.

During June 14–July 10, >11,000 phone calls were made to obtain 184 controls matched to 91 case-patients; case-patients with no matched controls were excluded from subsequent analyses (Figure 3). Because of differences in possible exposures (primarily the use and type of contact lens solutions) between soft lens, rigid lens, and non–contact lens users, we further restricted the analysis to case-patients (n = 72) and controls (n = 140) who reported wearing soft contact lenses only. Separate analyses were performed among users of rigid contact lenses and non–contact lens users; however, sample sizes were small, and no associations were found. Users of soft contact lenses who were excluded for lack of a matched control were not significantly different from those included in the analysis with respect to age, sex, race, and ethnicity.

Matched univariate analysis of users of soft contact lenses (Appendix Table) indicated that any use of AMOCMP within the month before symptom onset was a substantial risk factor (OR 15.8, 95% CI 5.6–44.6). No other contact lens solutions were associated with disease. Variables in univariate analyses that were included in the multivariate modeling included the following: any use of AMOCMP, Hispanic ethnicity, age (12–17, 18–24, 25–34, 35–49, versus >50 years), male sex, history of ocular trauma, contact lens use <5 years, frequency of replacing old lenses with new ones, swimming in a lake or river while wearing lenses, washing the face while wearing lenses, lack of hand washing before inserting lenses, cleaning lenses at the bathroom sink, failure to always cap the solution bottle after use, and ever topping off solution (adding new solution to old solution in the lens case).

In multivariate analysis, only 3 exposures were statistically significant (Table 2). Case-patients had 16.9 times the odds of reporting any use of AMOCMP compared with controls (95% CI 4.8–59.5), 2.8 times the odds of reporting ever topping off solution (95% CI 1.2–6.8), and 2.8 times the odds of having used contact lenses for <5 years (95% CI 1.0–7.6). Although age and sex were not significantly associated with AK in the multivariate analysis, they were included in the model to adjust for potential confounding.

**Table 2:** Multivariate analysis of demographic characteristics and exposures among 72 *Acanthamoeba* keratitis case-patients and 140 controls who used soft contact lenses, United States, 2005–2007*

Demographic characteristic or exposure†	No. (%) case-patients, n = 72	No. (%) controls, n = 140	Adjusted matched OR (95% CI)‡
AMOCMP	40 (55.6)	15 (10.7)	16.9 (4.8–59.5)
Ever top off or reuse old solution in case	40 (57.1)	30 (22.7)	2.8 (1.2–6.8)
<5 y as contact lens wearer	37 (51.4)	32 (22.9)	2.8 (1.0–7.6)

*OR, odds ratio; CI, confidence interval; AMOCMP, Advanced Medical Optics Complete MoisturePlus. †During 1mo before symptom onset. Missing values excluded from analysis. ‡Adjusted for age and sex. p<0.05.

### Laboratory Investigation

We received 94 outbreak–related specimens from case-patients; 10 culture isolates, 4 corneal specimens, and 80 environmental specimens (48 lenses and/or cases, 32 bottles of solution, including 5 unopened bottles). Of the 4 corneal specimens, 1 (25%) was culture positive; that specimen and 2 others (overall 75%) were positive by real-time PCR. Among the 48 lenses and/or cases, 11 (23%) were positive by culture; real-time PCR detected an additional 3 positive samples (overall 29%). No bottles of solution were culture positive. Five opened AMOCMP bottles were positive by real-time PCR. Eight other AMOCMP bottles, including 3 that arrived in the laboratory unopened, were negative by PCR. The remaining bottles of solution (non-AMOCMP products) were all PCR negative. Of 22 different lot numbers for AMOCMP bottles used by case-patients before onset of symptoms, no lot number was repeated.

*Acanthamoeba* genotyping was performed on 22 outbreak–related isolates; 20 (91%) were T4 genotype, which is the most common genotype in the environment as well as in AK (*11*). Two environmental samples contained *Acanthamoeba* genotypes T3 and T14.

## Discussion

This investigation of a national AK outbreak identified use of a single contact lens solution as the primary risk factor for infection. AMOCMP is a multipurpose solution used for disinfecting, rinsing, cleaning, and storing lenses. Our findings indicate that the strong association between AMOCMP and AK was unlikely to have resulted from intrinsic contamination. Case-patients had a wide geographic and temporal distribution, and there were no common lot numbers among AMOCMP bottles provided by case-patients. Although 3 opened bottles of AMOCMP tested positive by PCR for *Acanthamoeba* spp., all unopened bottles were PCR negative, and all AMOCMP bottles were negative by culture. The positive PCR results most likely represent point-of-use contamination through contact with water or dirty hands. We suspect that AMOCMP was insufficiently active against *Acanthamoeba* spp., resulting in increased likelihood of infection among product users. The sources of *Acanthamoeba* spp. could have been varied and multiple because *Acanthamoeba* spp. are ubiquitous in the environment. A concurrent AK case–control study conducted in the Chicago area, which investigated 55 separate cases, also found AMOCMP to be the most important risk factor (*9*).

AMOCMP was introduced for sale in 2003, just before the nationwide increase in cases. This product differed from AMO Complete, the multipurpose solution that preceded it, by the addition of propylene glycol (a comfort enhancer) and taurine (a buffering agent) (*12*). After this outbreak, laboratory investigations found that propylene glycol may cause *Acanthamoeba* trophozoites to encyst, thereby making them resistant to disinfection (*12**,**13*). However, propylene glycol is not unique to AMOCMP and is an ingredient of at least 1 other multipurpose solution, as well as several brands of artificial tears. Published results on the performance of multipurpose solutions, including AMOCMP, against *Acanthamoeba* organisms have shown varying efficacy (*14**–**16*). Assessment of anti-*Acanthamoeba* solution efficacy is limited by a lack of standardized testing methods (*17*). FDA guidance and the International Organization for Standardization standards do not include anti-*Acanthamoeba* spp. testing (*18*). However, after this outbreak, the Ophthalmic Devices Panel of the FDA Medical Devices Advisory Committee has recommended adding *Acanthamoeba* spp. as a challenge organism for testing of contact lens solutions (*19*).

Several similarities exist between this AK outbreak and the 2006 *Fusarium* keratitis outbreak (*7*). Both outbreaks of serious corneal infections occurred primarily among users of soft contact lenses. The 3–4 year duration of the AK outbreak spanned the timeframe of the *Fusarium* outbreak (June 2005–June 2006). In each outbreak, the primary risk factor was use of a certain multipurpose solution; Bausch and Lomb Renu with MoistureLoc (RML) was recalled in April 2006 after its use was identified as a major risk factor for *Fusarium* keratitis. Neither investigation found evidence of intrinsic microbial contamination of the solution; instead, insufficient antimicrobial efficacy was hypothesized to be the primary driving force behind each epidemic. In both outbreaks, the practice of topping off solution in the lens case also emerged as an important risk factor. After the *Fusarium* keratitis outbreak, tests simulating the reported practices of the case-patients found that topping off reduced the antimicrobial efficacy of RML (*20*). Together, these outbreaks raise concern about the safety of multipurpose contact lens solutions and related consumer behavior. FDA recently discussed these concerns and recommended changes to premarket testing and labeling for contact lens solutions, including more explicit warnings against topping off the solution (*19*).

This investigation yielded several notable negative findings. No use of contact lens solutions other than AMOCMP was identified as a risk factor, including the 1 other multipurpose solution that contained propylene glycol. Contact lens characteristics that have been hypothesized to increase the risk for AK, such as lens material and FDA lens group (*21**,**22*), were not associated with AK in this study; nor was there statistical interaction between use of AMOCMP and characteristics of soft contact lenses. Although rubbing and rinsing of lenses during the disinfection process have been shown in laboratory studies to decrease *Acanthamoeba* contamination of lenses (*23*), we did not find these practices to be protective. Although poor contact lens hygiene is widely recognized as a risk factor for AK (*24**–**26*), in this study only the topping off of old solution emerged as an important behavioral risk factor. We speculate that the association between AK and contact lens use for <5 years may reflect a wide range of poor hygiene practices among new contact lens users. Individual hygiene lapses may not be prevalent enough to emerge as important risk factors, but in aggregate, poor hygiene practices might be more common among users of new lenses.

We also found that a wide range of behaviors that can result in exposure of contact lenses to water (e.g., showering with lenses in, rinsing lenses or cases with tap water) was not associated with AK in this study. Although such practices are generally considered to be important risk factors for AK (*2**,**24**,**26*), a separate case–control study among contact lens users found that water exposure was not a risk factor (*27*).

Some researchers have suggested that municipal water treatment type may play a role in the development of AK (*28*). *Acanthamoeba* organisms are present in the water of many households (*29**–**32*). A temporal association was noted between an increase in AK cases in the Chicago area (*28*) and the implementation of the Environmental Protection Agency Disinfectants and Disinfection Byproducts Rule, which was aimed at decreasing potentially harmful disinfection byproducts in water (*33*). To comply with this rule, many municipal water supply systems have switched from using chlorine to using chloramine as a residual drinking water disinfectant. However, no change in water disinfection type was made by the Chicago Department of Water Management during the period of interest. Chicago-area water has been continuously disinfected with chlorine for >30 years (A. Stark, City of Chicago Department of Water Management, pers. comm.). A preliminary analysis (J. Verani, unpub. data) conducted during the early phase of this investigation found that only 12 (29%) of 41 case-patients for whom water treatment data were available received household water from chloraminated systems during the month before symptom onset, compared with an estimated 32% of the general US population (*34**,**35*). These findings suggested that water disinfection type was not an important risk factor in this outbreak.

This study had several limitations. First, because AK culture is a highly specific, but insensitive, diagnostic tool (*36*), and because preferred diagnostic methods vary by medical center, inclusion of only patients with culture-confirmed cases may have introduced regional testing bias and underestimated the scope of the outbreak. Second, the response rate among persons approached for control interviews was low; therefore, demographic differences between cases and controls may have been due to selection bias among controls. Third, recall bias may have been introduced as we asked participants to report on contact lens product use and behavior during the previous 2 years. Fourth, misclassification bias may have been introduced because at least 2 case-patients appeared to not differentiate between use of saline and cleaning or disinfecting solutions. Fifth, because >40% of case-patients and all controls were interviewed after AMOCMP was recalled in May 2007, reporting bias may have been introduced.

Despite these limitations, among users of soft contact lenses, case-patients had almost 17 times the odds of reporting any AMOCMP use compared with matched controls, validating the results of the preliminary analysis comparing AK cases to *Fusarium* keratitis investigation controls (*8*). The use of this existing *Fusarium* comparison data enabled rapid public health action months before the case–control study was completed. Recent associations of 2 distinct multipurpose solutions with outbreaks of rare corneal infections highlight the need for improved surveillance to promptly detect contact lens–related outbreaks and raise concerns about the effectiveness of multipurpose solutions. Continued monitoring of AK case trends to assess the impact of the AMOCMP recall and research on the anti-*Acanthamoeba* efficacy of AMOCMP and other solutions are under way. Our findings highlight the importance of promoting healthy habits among contact lens users, particularly discouraging the practice of topping off solutions and reinforcing safe hygienic practices among new users of contact lenses, as well as the need for standardized anti-*Acanthamoeba* testing of contact lens solutions.

## Supplementary Material

Appendix Table Univariate analysis of demographic characteristics and exposures among 72 Acanthamoeba keratitis case-patients and 140 controls who used soft CLs, United States, 2005-2007*
