# Supplementary material for: National Outbreak of Acanthamoeba Keratitis Associated with Use of a Contact Lens Solution, United States
Source: Emerg Infect Dis. 2009 Aug;15(8):1236–42. doi: 10.3201/eid1508.090225 (PMC2815976; doi:10.3201/eid1508.090225)
Supplement: Appendix Table — Univariate analysis of demographic characteristics and exposures among 72 Acanthamoeba keratitis case-patients and 140 controls who used soft CLs, United States, 2005-2007* [file 09-0225_appT-s1.pdf]

Appendix Table. Univariate analysis of demographic characteristics and exposures among 72 *Acanthamoeba* keratitis case-patients and 140 controls who used soft CLs, United States, 2005–2007*

Demographic characteristic or exposure†‡	No. case-patients (%), n = 72	No. controls (%), n = 140	Unadjusted matched odds ratio (95% CI)
Age, y			
12–17	23 (11.1)	9 (4.3)	7.4 (2.4–23.1)§
18–24	13 (6.2)	11 (5.3)	3.7 (1.2–11.6)§
25–34	9 (4.3)	26 (12.5)	1.0 (0.3–3.1)
35–49	13 (6.2)	52 (25.0)	0.8 (0.3–2.1)
≥50	14 (6.7)	38 (18.3)	ref
Male sex	28 (38.9)	30 (21.6)	2.4 (1.3–4.6)§
Hispanic ethnicity	11 (15.3)	7 (5.2)	12.2 (1.5–99.5)§
White race (vs. non-white)	62 (88.6)	126 (91.3)	0.6 (0.2–2.1)
Medical history			
Smoker, ever	23 (31.9)	30 (21.6)	1.6 (0.8–3.0)
Diabetes	2 (2.8)	4 (2.9)	1.0 (0.1–8.0)
Immunosuppresion	1 (1.4)	2 (1.4)	1.0 (0.1–11.0)
Ocular trauma	8 (11.1)	5 (3.6)	4.6 (1.2–17.8)§
CL type†¶			
FDA group 1	16 (24.6)	26 (22.4)	1.5 (0.7–3.2)
FDA group 2	2 (3.1)	19 (16.4)	0.2 (0.1–1.1)
FDA group 3	2 (3.1)	3 (2.6)	1.2 (0.2–7.7)
FDA group 4	45 (69.2)	68 (58.6)	1.1 (0.6–2.3)
Silicone hydrogel lens	16 (25.0)	27 (21.8)	1.5 (0.7–3.3)
Surface-treated lens	7 (10.9)	10 (8.1)	1.6 (0.6–4.8)
CL use†			
CL to correct vision	72 (100)	136 (98.6)	–
≤5 y as CL wearer	37 (51.4)	32 (22.9)	3.8 (1.9–7.6)§
Daily-wear CLs#	58 (81.7)	105 (75.0)	1.5 (0.8–3.0)
Daily disposable CLs**	1 (1.4)	3 (2.19)	0.5 (0.1–5.4)
Wear ≥5 d per week	67 (93.1)	118 (84.3)	2.3 (0.8–6.2)
Wear ≥12 h per day	48 (81.4)	96 (86.5)	0.8 (0.4–1.8)
Ever sleep overnight with CL	17 (23.6)	45 (32.1)	0.6 (0.3–1.3)
CL replacement interval >1 mo	6 (8.6)	31 (22.6)	0.3 (0.1–0.9)§
CL disinfection, cleaning, and hygiene†			
Do not always wash hands before cleaning CL	22 (33.3)	35 (28.0)	1.2 (0.6–2.3)
Clean at bathroom sink††	38 (35.6)	56 (47.9)	0.5 (0.2–1.0)§
Do not rub CL during cleaning	27 (40.9)	44 (34.9)	1.4 (0.7–2.5)
Do not rinse CL during cleaning	22 (33.3)	31 (25.0)	1.7 (0.8–3.5)
Rinse for <5 s for each side of CL	20 (52.6)	45 (51.1)	0.9 (0.4–2.1)
Do not always wash hands before inserting CL in eyes	25 (35.2)	30 (21.6)	1.9 (1.0–3.6)§
Ever handle CL with wet hands	38 (53.5)	74 (53.2)	1.1 (0.6–1.9)
Do not always cap solution bottle when finished	4 (5.7)	33 (24.3)	0.2 (0.1–0.6)§
CL care products used†			
Saline solution	7 (9.7)	23 (16.4)	0.6 (0.2–1.3)
Daily cleaner	6 (8.3)	10 (7.4)	1.2 (0.4–3.7)
Multipurpose solution	63 (87.5)	119 (85.0)	1.2 (0.6–2.8)
AMOCMP‡‡	40 (55.6)	15 (10.7)	15.8 (5.6–44.6)§
Hydrogen peroxide solution	4 (5.6)	7 (5.0)	1.1 (0.3–3.9)
Conditioning or wetting solution	1 (1.4)	4 (2.9)	0.6 (0.1–5.2)
Rewetting drops of artificial tears	13 (18.1)	18 (12.9)	1.5 (0.7–3.3)
Heating or ultrasonic device	1 (1.4)	1 (0.7)	2.4 (0.2–39.7)
Enzymatic cleaner or protein remover	2 (2.8)	6 (4.3)	0.7 (0.1–3.5)
CL storage practices (ever vs. never)†			
Top off or reuse old solution in case	40 (57.1)	30 (22.7)	4.4 (2.2–8.5)§
Store CL in tap water	3 (4.3)	10 (7.5)	0.5 (0.1–2.1)
Store CL ≤12 h in case	60 (85.7)	111 (84.1)	1.1 (0.5–2.4)
Leave case open while storing CL	0 (0)	8 (6.1)	–
Rinse case >1 time per week	17 (24.3)	40 (30.3)	0.8 (0.4–1.5)
Clean case with tap water	45 (62.5)	73 (52.1)	1.4 (0.8–2.4)
Use the same CL case for >6 mo	38 (58.5)	85 (68.6)	0.7 (0.4–1.3)

Water exposure while wearing CL (ever vs. never)†			
Swim, any location	28 (38.9)	45 (32.4)	1.3 (0.7–2.5)
Swim in lake or river	9 (12.5)	6 (4.3)	3.8 (1.2–12.7)§
Swim in pool	23 (31.9)	38 (27.1)	1.2 (0.6–2.5)
Swim in ocean or salt water	9 (12.5)	16 (11.4)	1.0 (0.4–2.6)
Use hot water spa or jacuzzi	12 (16.9)	39 (28.3)	0.5 (0.3–1.1)
Participate in water sports	9 (12.7)	8 (5.9)	2.3 (0.8–6.7)
Shower	57 (79.2)	106 (76.3)	1.2 (0.6–2.4)
Take bath	16 (22.2)	49 (35.2)	0.5 (0.3–1.0)
Wash face	42 (59.2)	108 (77.7)	0.4 (0.2–0.8)§

*CL, contact lens; CI, confidence interval; FDA, Food and Drug Administration; AMOCMP, Advanced Medical Optics Complete MoisturePlus.
†During 1 mo before symptom onset of case-patient.
‡Missing values excluded from analysis.
§$p<0.05$.
¶Tyler's Quarterly Soft Contact Lens Parameter Guide. 2006;23(2) and 2007; 24(2).
#Compared with extended wear.
**Compared with nondaily disposable contact lenses.
††Compared with in the bathroom, but not at the sink.
‡‡No other multipurpose solution was reported to be used by >5 case-patients during the month before symptom onset.
